# Sow behaviour, parturition process and management in free farrowing systems

**DOI:** 10.1186/s40813-026-00505-5

**Published:** 2026-03-31

**Authors:** Alexander Grahofer

**Affiliations:** Clinic for Swine, Department of Clinical Veterinary Science, Vetsuisse Faculty, University of Bern, Bremgartenstrasse 109a, Bern, 3012 Switzerland

## Abstract

The pig industry is increasingly transitioning from traditional farrowing crates to free farrowing systems, driven by growing concerns for animal welfare and regulatory pressures to enhance sow well-being. Free farrowing systems, which allow sows greater freedom of movement, have emerged as a welfare-oriented alternative to traditional farrowing crates. These systems enable the expression of natural behaviours, particularly nest-building, which is hormonally driven and peaks shortly before parturition. Adequate provision of diverse nesting materials facilitates nest-building, optimizes endocrine preparation for farrowing, and can shorten farrowing duration, improving piglet vitality and colostrum intake. Farrowing in sows encompasses three stages: preparatory behaviour and cervical dilation, active piglet expulsion, and placental expulsion. Free farrowing systems are associated with lower maternal stress, higher endogenous oxytocin, and more natural behavioural expression, potentially influencing parturition kinetics. However, dystocia incidence remains comparable to crated systems, indicating that careful management is still required. Key indicators for monitoring farrowing in free farrowing sows include piglet birth intervals, placental expulsion timing, litter-level meconium scoring, umbilical cord integrity, and maternal physiological markers such as progesterone. Excessive or routine administration of exogenous uterotonics is discouraged due to the risk of overly strong contractions, increased stillbirths, and reduced colostrum intake. Placental expulsion patterns offer additional insights, with sows expelling the first placenta before or alongside the last piglet, or with more than four placental parts, exhibiting prolonged farrowing. While free farrowing systems improve welfare and allow more natural parturition, effective management based on sow behaviour and physiology remains essential to mitigate risks and optimize reproductive outcomes. In conclusion, free farrowing systems represent a significant advancement in sow welfare, facilitating natural maternal behaviours and promoting better physiological preparation for parturition. Continued research is needed to refine monitoring strategies, define intervention thresholds, and fully harness the potential of these systems to enhance both sow and piglet health, demonstrating that improved welfare can coexist with productive performance.

## Introduction

Reproductive performance is a key determinant of productivity and sustainability in modern pig production. In recent decades, genetic selection for hyper prolific sows has substantially increased litter size, but this progress has also been accompanied by a growing prevalence of farrowing-related and other reproductive disorders [1, 2]. These challenges are particularly evident during the peripartum period, when sows experience intense physiological and behavioural demands. Reproductive disorders are among the most common and economically significant problems in breeding herds and represent the leading cause of sow culling [3, 4]. Reported prevalence rates of peripartum reproductive disorders vary widely, ranging from 29.6% [5] and 33.3% [6] to as high as 71.0% [7], underscoring both the magnitude of the problem and differences in management, housing, and assessment criteria. This variability highlights the need for a more refined understanding of the parturition process itself, including how it is influenced by environmental conditions and how deviations from normal progression should be defined and measured. Within this context, animal welfare considerations have become increasingly prominent. The fourth freedom for animals, as defined in the “Five Freedoms” of animal welfare, is the freedom to express normal behaviour [8]. This freedom underscores the necessity of providing animals with an environment that allows them to perform species-specific behavioural patterns essential for their health and welfare. For periparturient sows, these behaviours include nest-building, nursing, and maternal interactions with piglets. Sows exhibit a strong, hormonally driven motivation to perform nest-building behaviour before farrowing, which typically ceases by the time of parturition [9–12]. This behaviour involves exploring the environment and gathering suitable materials to prepare a nest, which provides comfort and protection for parturition and the newborn piglets. To ensure the expression of these natural behaviours, sows require specific environmental conditions. Adequate space allows sows to move freely, turn, and engage in nest-building activity [9, 10, 13, 14]. Furthermore, access to suitable substrate, such as straw or other manipulable materials, is crucial for satisfying nest-building motivation and reducing stress levels [9, 10, 15–17].

Normal parturition in the sow is regulated by a tightly coordinated hormonal cascade involving declining progesterone concentrations, increasing oestrogens and prostaglandins, and the release of oxytocin, which together initiate uterine contractions, foetal expulsion, and placental release [18–20]. Disruptions to this endocrine balance may result in prolonged farrowing, incomplete placental expulsion, or increased susceptibility to postpartum inflammatory disorders [1, 2, 4, 21–25]. Despite the importance of these processes, practical on-farm assessment of parturition quality often relies on indirect indicators, such as farrowing duration, birth intervals, and stillbirth rates, rather than direct evaluation of uterine function or placental expulsion. Housing conditions during farrowing may further influence the physiological and behavioural expression of parturition. Adequate space, freedom of movement, and access to manipulable materials can affect sow activity patterns, stress responses, and possibly the endocrine regulation of labour [9–12]. In this context, free farrowing systems have been proposed as an alternative to conventional crates, as they allow greater behavioural flexibility during the periparturient period. However, evidence regarding their effects on the progress of parturition, sow health, and farrowing-related disorders remains fragmented and, in some cases, contradictory.

The pig industry is increasingly shifting from traditional farrowing crates to free farrowing systems [26, 27]. This change is driven by growing concerns about animal welfare and regulatory demands to improve sow well-being by allowing greater freedom of movement and the expression of natural behaviour [27–29]. From a regulatory perspective, several countries have already taken significant steps toward free farrowing systems. Farrowing crates have been prohibited in Sweden (since 1987), Switzerland (since 1997), and Norway (since 2000) [27]. At the European level, the European Commission’s animal welfare strategy aims to ensure that pigs are housed without crates by 2027 [30]. However, this deadline has been postponed once again, highlighting the ongoing challenges of reconciling animal welfare objectives with production efficiency [31]. Meanwhile, Austria, Germany and Denmark have enacted legislation to gradually phase out the permanent use of farrowing crates, with deadlines set for 2033, 2036 and 2041, respectively [27, 32].

While the transition from farrowing crates to free farrowing systems is often discussed from an animal welfare perspective, less attention has been given to how parturition itself should be evaluated under these systems. Differences in sow posture, activity, and interaction with the environment may alter commonly used indicators of farrowing difficulty, complicating comparisons with conventional housing. Consequently, there is a need to critically assess whether existing definitions, measurement approaches, and thresholds for abnormal parturition are appropriate for free farrowing systems.

The aim of this review is therefore to synthesise current knowledge on sow behaviour and physiology during the periparturient period in free farrowing systems, with particular emphasis on the parturition process itself. Specifically, this review examines how housing and management practices influence hormonal regulation, uterine activity, placental expulsion, and the interpretation of farrowing outcomes. By identifying key gaps in knowledge and methodological challenges, this review seeks to support the development of management strategies and assessment tools that optimise both reproductive performance and sow welfare in free farrowing systems.

## Nest-building behaviour in a free farrowing system

Free farrowing systems provide sows with greater opportunities to express their natural behaviours and enhance locomotion during the peri- and postpartum periods compared with crates, which positively affects sow health and welfare. [9, 10, 27, 28, 33–35]. One significant advantage of these systems is that they allow sows to express their nest-building behaviour, with higher activity levels observed compared to crated sows [13, 35–38].

Nest-building is an intrinsic behaviour that coincides with hormonal changes, initiated by increased prolactin concentrations following a decline in progesterone and a rise in prostaglandin F2α [9, 10, 33, 38].

The sow initiates nest-building approximately 24 hours before farrowing, with peak activity occurring 4 to 12 hours before the birth of the first piglet [11, 35]. This behaviour ceases as oxytocin concentrations rise and udder comfort improves, due to piling of nest-building material [10, 39–41]. Prolonged nest-building behaviour, maintained during parturition, may be considered abnormal, and it appears indicative of problems in the hormonal process of parturition [10]. It is well-established that insufficient or unsuitable nesting materials during the periparturient period can cause stress in sows, leading to increased opioid release [10]. This, in turn, suppresses oxytocin secretion, reducing uterine contractility [41–43]. Hence, encouraging natural nesting behaviour can help shorten farrowing duration and thereby improve piglet vitality and enhance colostrum intake [10, 40, 44–47].

Although various types and amounts of nest-building materials influence the farrowing process in confined systems, there is limited research on their effects in free farrowing systems. A recent study reported that sows in free farrowing systems predominantly used newspaper (one sheet twice daily) for nest-building, as indicated by the total duration of material manipulation, compared with straw (1 L twice daily) or jute fabric (0.5 m^2^) [48].

Providing sows with a diverse nest-building materials (two bucketfuls of sawdust, a shredded newspaper, three bucketfuls of chopped straw, seven branches of a tree, and three natural sisal ropes of 50 cm length) approximately 7 days before farrowing (information provided on request from first author) in a farrowing pen led to elevated plasma oxytocin concentrations in sows 3 days prior to parturition until 7 days postpartum compared with those housed in environments (crate or pen) with provision of a bucketful of sawdust. The least-squares means of oxytocin concentrations with standard error were: Diverse pen: 14.3 (1.1) pg/mL; Crate:11.2 (1.0) pg/mL; Pen: 11.1 (1.0) pg/mL; *p* = 0.037 [41]. A different study showed that sows provided with 2 kg of long-stemmed straw before parturition had shorter farrowing durations compared with sows given 4 kg of peat or no enrichment (295 min vs. 322 min and 438 min, respectively; *p* < 0.001) [44]. Moreover, the straw group had the lowest proportion of stillborn piglets compared with the peat and no enrichment groups (2.8% vs. 6.0% and 8.1%; *p* < 0.001) [44]. Interestingly, one study reported that larger litters (≥17 piglets) delayed the peak of nest-building activity in free farrowing sows to the onset of parturition, whereas sows with smaller litters (≤16 piglets) reached peak activity 8–6 hours before farrowing [49]. In conclusion, multiple factors influence nest-building behaviour in free farrowing sows, thereby affecting the farrowing process and its outcomes. Notably, substrate type appears to play a key role in shaping this behaviour. It may also be worth considering whether free-farrowing sows are more tolerant of different substrate types, as they are less likely to lose substrate through crate structures.

## Management of parturition in a free farrowing system

**Fig. 1:**
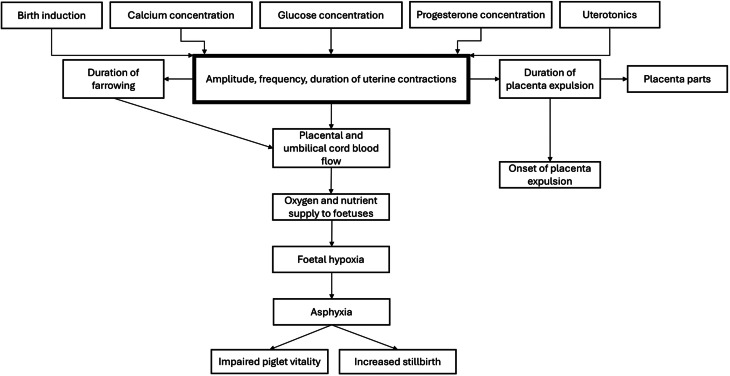
Relationship of maternal and management factors with uterine contractions, their association with the farrowing process in sows, and subsequent effects on offspring outcomes

As illustrated in Fig. 1, parturition in the sow is a complex physiological process regulated by endocrine, neural, and mechanical factors that culminate in the delivery of piglets and the expulsion of the foetal membranes [1, 2, 18]. It is traditionally divided into three stages [18]. Understanding the normal progression of parturition is essential for effective farrowing management, reduction of piglet mortality, and maintenance of sow health.

### Farrowing process

Stage I of parturition is characterized by preparatory events leading to cervical dilation and positioning of the foetuses for delivery [18, 50–52]. This stage may last from 2 to 24 hours and is marked by behavioural changes such as restlessness, nest-building activity, reduced feed intake, and an increased frequency of postural changes [1, 2, 10, 29, 37, 38]. Physiologically, uterine contractions become more coordinated and cervical relaxation occurs under the influence of hormonal changes, particularly declining progesterone concentration, rising concentrations of oestrogen and prostaglandins, and increased oxytocin receptor expression. [18, 19].

**Fig. 2:**
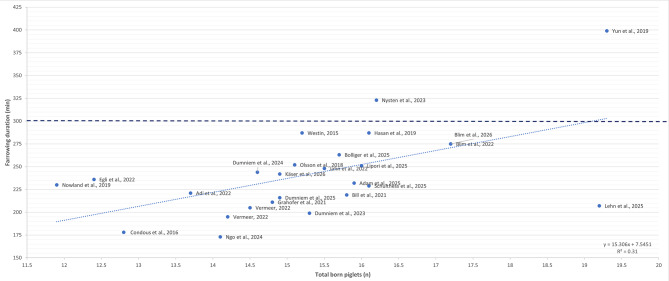
Overview of farrowing duration and total number of piglets born in free farrowing sows over the past ten years [23, 45, 49, 53–70, 73, 74]. The dashed line indicates the reference value for prolonged farrowing [13], and the dotted line shows the linear regression between farrowing duration and total born piglets

Stage II involves the active expulsion of foetuses and is the most critical phase of farrowing. Coordinated myometrial contractions and abdominal straining facilitate piglet delivery, with reported mean piglet-piglet intervals in free farrowing systems ranging from 14 to 17 minutes and individual intervals exhibiting substantial variability (3 - 154 minutes) [42, 53, 54]. The total duration of this stage in free farrowing systems averages 230 minutes (range: 173–399 minutes; Fig. 2) [45, 49, 53–71]. Efficient progression of piglets birth depends on adequate uterine contractility and minimal stress. These conditions appear to be better supported in free-farrowing sows, which exhibit higher oxytocin concentrations than crated sows [60, 72]. Post-expulsion oxytocin pulses tended to be higher from sows in a free farrowing system (77.6 ± 47.6 pg/ml) compared to crated sows (38.1 ± 24.6 pg/ml; *p* = 0.08) [72].

Stage III consists of the expulsion of the placental membranes. Complete expulsion of the placenta usually occurs within a few hours postpartum in crated sows [18, 24, 75]. Several studies indicate that placental expulsion duration is longer in free farrowing systems compared to crated sow, with an average of 313 min (Table 1) [24, 53, 54, 59]. Further details regarding these characteristics are provided in the section on placental traits. 

Retained placental tissue is uncommon in swine but may predispose affected animals to postpartum disorders such as metritis [21, 22, 75–78]. However, data on its occurrence in free farrowing sows are currently lacking.

**Table 1:** Incidence of dystocia in sows housed in free farrowing and conventional crated systems, as reported over the past decade [23, 45, 49, 53–70, 73, 74], based on various definitions of prolonged piglet birth intervals, sorted in ascending order of incidence. Free farrowing data are highlighted in bold

Reference	Definition (piglet-piglet interval)	Farrowing system	incidence (%)
Zaremba et al., 2019 [79]	>45 min	Crated	11
Feyera et al., 2018 [80]	>90 min	Crated	24
Dumniem et al., 2025 [56]	>45 min	**Free farrowing**	**22**
Dumniem et al., 2025 [56]	>45 min	Crated	23
**Bill et al., 2021** [53]	**>60 min**	**Free farrowing**	**29**
Wongwaipisitkul et al., 2024 [81]	>30–60 min	Crated	30
**Grahofer et al., 2021** [55]	**>60 min**	**Free farrowing**	**40**
**Blim et al., 2022** [82]	**>60 min**	**Free farrowing**	**40**
Nagi Dorio et al., 2023 [83]	>60 min	Crated	40
**Käser et al., 2026**	**>60 min**	**Free farrowing**	**42**
Nam & Sukon 2021 [84]	>45 min	Crated	47
**Nystén et al., 2023** [49]	**>90 min**	**Free farrowing**	**48**
**Schulthess et al., 2025** [62]	**>60 min**	**Free farrowing**	**49**
Nam et al., 2022 [85]	>45 min	Crated	50
Adi et al., 2024 [86]	>45 min	Crated	51
**Bolliger et al., 2025** [57]	**>60 min**	**Free farrowing**	**53**
Blim et al., 2022 [82]	>60 min	Crated	55
**Egli et al., 2022** [66]	**>60 min**	**Free farrowing**	**56**
Blim et al., 2026	>60 min	Crated	56
**Jahn et al., 2022** [54]	**>60 min**	**Free farrowing**	**58**
**Lehn et al., 2025** [60]	**>60 min**	**Free farrowing**	**60**
**Adam et al., 2025** [59]	**>60 min**	**Free farrowing**	**60**
**Blim et al., 2026**	**>60 min**	**Free farrowing**	**60**
Lehn et al., 2025 [60]	>60 min	Crated	67
Haller et al., 2025 [87]	>60 min, >45 min, >30 min	Crated	71

Several studies have demonstrated that farrowing duration is shorter in free farrowing sows compared to crated sows [42, 60, 82]. This difference may be attributed to the inhibitory effects of elevated opioid concentrations and a higher density of opioid receptors in crated sows, which are thought to suppress endogenous oxytocin release during parturition [42]. A summary of validated scientific data on farrowing durations in free farrowing sows in relation to total born piglets, collected over the past ten years, is presented in Fig. 2.

Additionally, two studies found that housing conditions influence hormonal regulation during farrowing [60, 63]. Free farrowing sows exhibited lower intrapartum progesterone concentrations compared to crated sows [60, 63]. Although one study did not perform formal statistical analysis, its descriptive data suggested a comparable trend [63], whereas another study identified a statistically significant difference (*p* = 0.046) [60]. This difference may be associated with the significantly lower concentrations of prostaglandin F2α metabolite observed in crated sows, suggesting a potential impact of confinement on the hormonal regulation of parturition [60]. 

### Prolonged farrowing/dystocia

**Fig. 3:**
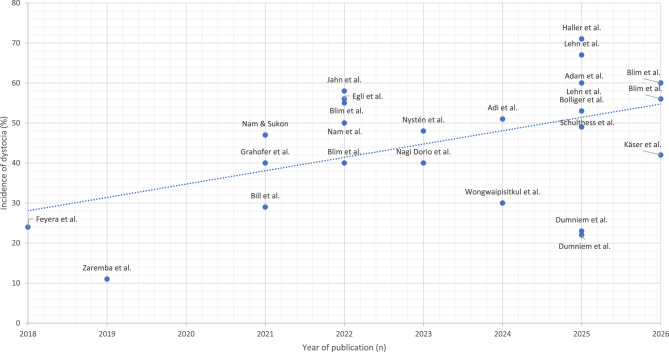
Increase in the incidence of dystocia in sows housed in free-farrowing systems and conventional farrowing crates over the past decade [23, 45, 49, 53–70, 73, 74]. The dotted line represents the linear regression illustrating the relationship between dystocia incidence and year of publication

Over the past ten years, the incidence of dystocia has significantly increased as shown in Fig. 3. Although free farrowing systems enable sows to express natural behaviours and influence hormonal changes during the peripartal period, the incidence of dystocia over the last ten years remains comparable to that observed in crated systems (free farrowing sows: 47.5% vs. crated sows: 43.8%), as presented in Table 1. Table 1 also provides an overview of dystocia incidence based on various definitions of prolonged piglet-piglet intervals. Hence, adequate management procedures are required to reduce and/or immediately recognize birth disorders also in free farrowing sows. Limited information is available in the literature on piglet–piglet intervals in sows housed in free farrowing systems. As mention earlier, reported mean intervals range from 14 to 17 minutes, with individual intervals varying from 3 to 154 minutes [42, 53, 54]. One study compared the piglet–piglet intervals between crated sows and sows housed in farrowing pens provided with different types and amounts of nest-building material. In the free-farrowing groups, one group received a single bucket of sawdust (limited material), whereas the other received a combination of saw dust, shredded newspaper, chopped straw, tree branches and sisal ropes (diverse material). The study indicated a trend (*p* = 0.07) towards a reduced piglet-piglet interval between the first and fifth piglets in crated sows (17 min) compared to free farrowing sows, both with limited (30 min) and with diverse nest-building material (28 min) [37]. Specifically, the mean farrowing duration from the first to the fifth piglet was shorter in crated sows (67 ± 15 min) compared with free farrowing sows provided with limited (119 ± 14 min) or diverse (104 ± 17 min) nest-building material, with a tendency toward statistical significance (*p* = 0.053). The mean farrowing duration from the first to the tenth piglet differed significantly among treatments (*p* = 0.044), being shortest in crated sows (114 ± 19 min), longest in free farrowing sows with limited nest-building material (180 ± 17 min), and intermediate in those provided with extensive nest-building material (170 ± 21 min) [37]. One plausible explanation for these observations lies in differences in sow behaviour during parturition. Free farrowing sows consistently spend a greater proportion of time standing, changing posture, and engaging in locomotor activity before and during farrowing, whereas crated sows are largely confined to lying or sitting positions. In natural or semi-natural environments, sows commonly alternate between standing and lying during parturition, particularly during the early stages, and posture changes are considered part of the normal behavioural repertoire associated with foetal expulsion. Increased standing and locomotion in free farrowing systems may therefore reflect a more natural expression of parturition behaviour rather than a pathological delay [36, 37]. Sows housed in crates exhibited significantly less exploratory behaviour at 6 and 3 hours before the onset of farrowing compared with sows in free farrowing systems with and without nest-building material (−6 h: 1.9% vs. 7.7% and 15.9%; −3 h: 2.6% vs. 7.3% and 16.5%; *p* < 0.05), whereas lying and sitting behaviours were more prevalent [36]. In addition, during the farrowing of the first five piglets, free farrowing sows exhibited significantly more standing and locomotor behaviour (pens with limited nest-building material: 19.2 min; pens with extensive nest-building material: 10.7 min) compared with crated sows (2.5 min; *p* < 0.05) [37]. Importantly, these behavioural differences raise critical questions regarding the suitability of reference values for piglet-piglet intervals and total farrowing duration that were originally developed under crated conditions. Applying crate-based thresholds to free farrowing systems may increase the risk of misclassifying physiologically normal variation as dystocia or delayed farrowing. This, in turn, could prompt unnecessary management interventions, such as premature obstetrical assistance or iatrogenic induction, which may disrupt the endocrine regulation of parturition and increase the risk of postpartum disorders.

Taken together, existing current evidence suggests that while faster farrowing is frequently reported in crated sows, this finding is neither universal nor straightforward to interpret. Rather than viewing longer or more variable farrowing durations in free farrowing systems as inherently problematic, there is a need to critically re-evaluate how parturition is assessed in these environments. Future research should focus on establishing housing-specific reference values that account for behavioural freedom and natural postural changes, thereby supporting management decisions that safeguard both sow health and the physiological integrity of the farrowing process. 

### Pen features

The focus of free farrowing systems is to enable sows to fulfil their biological needs throughout farrowing and lactation, promoting both welfare and productivity [33]. In a recent study in Sweden, 51 pen types were assessed, revealing substantial variation in pen dimensions, with lengths ranging from 259 to 415 cm (315.1 ± 24.3 cm), widths from 188 to 245 cm (207.0 ± 10.7 cm), and sow-available areas (total pen area minus piglet corner) ranging from 3.9 to 6.4 m^2^ (5.4 ± 0.6 m^2^) [88]. In addition, measurements of 146 sows showed body lengths of 129–238 cm (191.3 ± 19.3 cm) and heights of 74–133 cm (86.7 ± 7.7 cm), reflecting substantial variability in sow dimensions relevant to pen design [88]. These findings underscore the essential need for free farrowing pens to be designed to accommodate variation in sow, litter, and piglet size.

**Fig. 4:**
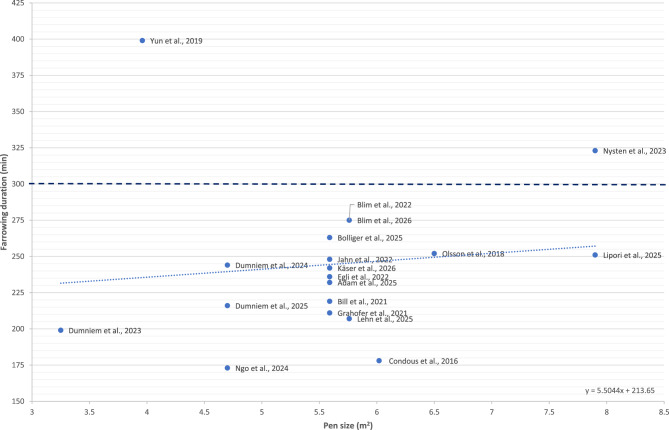
Overview of farrowing duration and pen size in free farrowing sows over the past ten years [23, 45, 49, 53, 54, 56–62, 64, 66, 69, 70, 73, 74, 82]. The dashed line indicates the reference value for prolonged farrowing [13], and the dotted line shows the linear regression between farrowing duration and pen size

A factor that remains poorly understood is the potential influence of pen design, including pen size, on farrowing duration. To date, only one study has evaluated two farrowing pen sizes (8 m^2^ vs. 24 m^2^) using the same genetic line and management conditions, reporting no significant difference in farrowing duration between pen sizes (8 m^2^ : 205 min vs. 24 m^2^ : 195 min; *p* = 0.60) [64]. Accordingly, based on research data collected from the past decade for this review, farrowing duration was analysed with respect to pen size, revealing no significant effect (Fig. 4). Currently, there is insufficient evidence to determine whether pen size significantly affects farrowing duration, and further research is warranted. 

Moreover, research has evaluated differences in air quality between free farrowing pens and conventional farrowing crates [82, 88]. Air quality (concentration of total airborne bacteria, haemolytic streptococci, molds, or yeast) between pen types has been similar between free farrowing pens and crates, suggesting existing ventilation and hygiene routines can be maintained when transitioning to free farrowing systems [82, 88].

In summary, current evidence suggests there is no singular, universally accepted minimum pen size for free farrowing that guarantees welfare outcomes. Assurance schemes and welfare bodies provide benchmarks (≈5–8 m^2^) [27, 89, 90] and some legislation sets specific minimum areas (e.g., 6.5 m^2^ in Germany). However, considerable variation in sow and piglet dimensions, litter behaviours, and design quality means that pen size interacts with layout, management, and design features to influence welfare outcomes. Recent reviews indicate that achieving mortality and welfare outcomes comparable to crate systems likely requires ≥ 7 m^2^ along with effective design and management. Nonetheless, space allowances may need to expand further as genetics and production practices evolve. Thus, the importance of pen size cannot be reduced to a mere factor related to the duration of parturition but must account for species‑typical behaviours and functional zones within the pen.

### Uterotonic agents

The routine use of uterotonic agents in crated sows has been reported to shorten farrowing duration and piglet-piglet interval, while increasing the average number of stillborn pigs [91, 92]. However, limited information is available regarding their use during parturition in free farrowing sows [53, 93, 94]. Two studies have evaluated the effects of routine oxytocin administration in free-farrowing sows [53, 93]. In one oxytocin was administered after the first piglet (20 IU < 130 kg; 30 IU for 130–180 kg; 40 IU < 250 kg; using two different oxytocin products, Oxy1 (Oxipar. Boehringer Ingelheim, Mexico) and Oxy2 (Biopar. Lapisa S. A. de C. V., Mexico)) [93]. In the other study, oxytocin (20 IU) was administered after the fourth piglet [53]. Neither study found a significant effect on farrowing duration [53, 93]. In contrast, oxytocin treatment was associated with a significant increase in intrapartum deaths compared with control sows, particularly between the first and fourth piglet (Control: 0.0% vs. Oxy1: 70.8% and Oxy2: 40.0%; *p* < 0.01) [93]. Additionally, oxytocin-treated sows exhibited significantly more severe meconium staining in stillborn piglets (Control: 0.10 ± 0.05 vs. Oxy1: 0.45 ± 0.11 and Oxy2: 0.50 ± 0.13; *p* < 0.05) and higher rates of ruptured umbilical cords (Control: 0.07 ± 0.05 vs. Oxy1: 0.42 ± 0.12 and Oxy2: 0.47 ± 0.12; *p* < 0.05) [93]. The incidence of dystocia was also significantly greater (*p* < 0.01) in oxytocin-treated sows [93]. In the second clinical study no significant difference between the control and the oxytocin-treated group on farrowing duration and other farrowing parameters were observed [53]. Recently a case report described severe adverse effects following the routine single intramuscular administration of 35 µg carbetocin at different time points during farrowing [94]. The routine treatment was linked to extended intervals between piglets (>60 min), excessive colostrum loss, and a higher incidence of weak and stillborn piglets [94]. Collectively, these findings suggest that the routine administration of oxytocin or its analogues is not recommended in free farrowing sows due to their potential negative effects. A possible explanation for these adverse outcomes is the significantly higher endogenous oxytocin concentrations observed in free farrowing sows during the post-expulsion phase of parturition compared to crated sows [13, 60, 82]. Therefore, administering additional exogenous uterotonic agents in this context may trigger excessively strong or prolonged uterine contractions, resulting in transient umbilical cord compression, compromised foetal oxygenation, and increased piglet mortality and colostrum loss, similar to the pathophysiological effects seen with oxytocin overdosing in confined sows [94, 95].

## Indicators of the quality of parturition for free farrowing systems

### Progesterone concentrations in blood and colostrum

The transition from gestation to farrowing is regulated by progesterone, which at high concentrations inhibits myometrial contractions [96]. Consequently, elevated progesterone concentrations at the onset of farrowing may influence both farrowing duration and farrowing outcomes [1, 2]. Although the relationship between progesterone concentrations and housing systems remains poorly understood, preliminary evidence suggests higher circulating progesterone concentrations on farms using farrowing crates (3.9 ng/mL) compared with a farm using a free farrowing system (1.6 ng/mL) [63]. In addition, a recent study that tested the housing system using the same genetic lines and under the same environmental conditions found significantly lower blood progesterone concentrations in sows housed in free farrowing system compared to crated sows [60]. Consistent with these findings, a subsequent study conducted in a free farrowing system reported that sows with higher blood progesterone concentrations at the onset of farrowing had a significantly higher incidence of stillbirths; specifically, sows with ≤1 stillborn piglet had lower blood progesterone concentrations than those with ≥2 stillborn piglets (1.47 ± 0.57 vs. 3.21 ± 1.93 ng/mL; *p* = 0.02) [49]. Furthermore, a study investigated progesterone concentrations in colostrum. Progesterone concentrations were positively correlated with log-transformed farrowing duration (*r* = 0.27; *p* = 0.03), piglet-placenta expulsion duration (*r* = 0.26; *p* = 0.04), and the incidence of severe meconium staining (*r* = 0.27; *p* = 0.04) [23]. In addition, sows requiring at least two obstetrical interventions had significantly higher progesterone concentrations in colostrum (25.9 ng/mL) compared with those requiring none or only one intervention (19.3 ng/mL; *p* = 0.02) [23]. Based on current knowledge, and despite the limited number of available studies, progesterone might be considered a potential indicator of the farrowing process in sows housed in free-farrowing systems. However, further research is needed to confirm the role as a marker of proper preparation for farrowing and to clarify its relationship with farrowing outcomes in this system.

### Blood glucose concentration

Farrowing is a physiologically demanding process that requires the sow to rapidly mobilize large amounts of energy to support uterine contractions and ensure successful delivery. Glucose is the primary energy substrate for the uterus [97], making the maintenance of sufficient blood glucose concentrations essential to meet the elevated energy demands during farrowing [98]. Sows in a free farrowing system exhibited lower blood glucose concentrations (4.4 mmol/L) than crated sows (5.5 mmol/L) [41, 42]. Although genetic factors and feeding management may influence glucose metabolism, it can be hypothesized that cortisol concentrations, known to be positively associated with glucose [99], may contribute to the observed reductions in blood glucose concentrations in free farrowing system. These findings are consistent with previous research showing that, compared with crated sows, two studies reported significantly lower cortisol concentrations prior to parturition in free-farrowing systems [100, 101], whereas four study found no significant difference between housing systems [42, 60, 102, 103], and one study, in contrast, reported significantly higher cortisol concentrations in free farrowing sows [104]. Such variability suggests that housing conditions may modulate cortisol secretion, which in turn could impact glucose metabolism around parturition. In addition, another possible explanation is that nest-building behaviour prior to farrowing increases glucose demand for uterine function. In free farrowing systems, sows are generally more active during the day [38], and this heightened activity may further elevate glucose metabolism, contributing to lower blood glucose concentrations at the onset of farrowing. In crated sows, higher blood glucose concentrations were associated with shorter farrowing durations, fewer stillbirths, and a reduced need for farrowing assistance [80, 105]. However, these relationships were not observed in sows in free farrowing systems. No significant correlation was detected between blood glucose concentrations at the onset of farrowing and farrowing duration, nor with stillbirths or the need for farrowing assistance [62]. This suggests that blood glucose concentrations at the onset of farrowing are not reliable predictors of prolonged farrowing (>300 min) or dystocia (>1-hour piglet-piglet interval) in free farrowing sows. Interestingly, older sows (≥5 litters) showed lower blood glucose concentrations at the onset of farrowing (4.3 ± 0.6 mmol/L vs. 4.6 ± 0.6 mmol/L; *p* = 0.02) and required more farrowing assistance (62% vs. 37%; *p* < 0.01), with nearly twice as many stillborn piglets compared to younger sows (1.42 ± 1.5 vs. 0.78 ± 1.5; *p* < 0.01) [62].

### Piglet traits

A significant interaction between farrowing duration and the number of stillborn piglets was observed in crated sows [106]. Facilitating natural behaviours has been shown to shorten farrowing duration, which can reduce the risk of stillbirths [34]. Overall, a meta-analysis found no significant difference in the number of stillborn piglets per litter between crated and free farrowing systems, although in 59% of the reviewed studies, a reduction in stillbirths was reported in free farrowing systems [14]. However, the relative risk of stillbirth was 22% higher in crated sows without enrichment materials compared to free farrowing sows without enrichment material [14]. Similar to crated sows, the occurrence of stillborn piglets in free farrowing systems can serve as an indicator of prolonged farrowing [68, 69]. Therefore, sows that expel a stillborn piglet during farrowing should be closely monitored for signs of dystocia. In addition, given the numerous risk factors for stillborn piglets, management practices such as improper use of uterotonic agents, and infectious causes should also be thoroughly assessed and ruled out in free farrowing farms [94]. Furthermore, a current study observed higher stillbirth rates in free farrowing sows with large litters (≥17 piglets; 10.9%) compared to those with small (≤13 piglets; 6.1%) or moderate (14–16 piglets; 2.8%) litter sizes (*p* < 0.001) [69]. One major limitation of the study was the routine administration of uterotonics (20 IU oxytocin) after the tenth piglet, which led to a significant increase in the proportion of stillborn piglets (3.4% vs. 14.7%, *p* < 0.001). However, as these findings were obtained in a free farrowing system and provide valuable context for its evaluation, they should be retained and critically considered in this review. Stillborn piglets exhibited significantly longer cumulative birth intervals (the interval between the birth of the first piglet and the birth of a given piglet) than live-born piglets (142.4 ± 9.35 min vs. 103.0 ± 3.71 min, *p* < 0.001), as well as higher body mass index (18.4 ± 0.39 vs. 17.1 ± 0.15 kg/m^2^; *p* = 0.002) and ponderal index (birth weight (kg)/(crown-rump length, m)^3^) (70.8 ± 1.59 vs. 63.7 ± 0.59 kg/m^3^; *p* < 0.001) [69]. Body-mass index and ponderal index can be assessed under scientific conditions with the ultrasound intra vitam [107]. However, they are not feasible for evaluation during on-farm farrowing, as they require advanced ultrasonographic expertise to accurately assess body parameters. Moreover, the procedure is time-consuming and may disturb the sow during parturition. Compared to body mass index and ponderal index, a more practically feasible indicator could be the meconium staining scores. Stillborn piglets showed increased meconium staining scores (2.04 ± 0.10 vs. 1.78 ± 0.04, *p* = 0.021) and a higher incidence of ruptured umbilical cords at birth (66.0% vs. 45.2%; *p* = 0.004) [69]. These data are essential for future research projects in which these parameters should be evaluated without human intervention, in order to better elucidate the association between farrowing characteristics (e.g., prolonged farrowing duration, late-born piglets) and stillbirth. At present, litter-level meconium scoring and the prevalence of ruptured umbilical cords after birth may serve as practical indicators for identifying sows with parturition disorders. This has also been discussed in previous studies, which reported that eutocic sows in free farrowing systems exhibited lower mean meconium scores than dystocic sows (0.73 ± 1.02 vs. 0.91 ± 0.99) [73]. Furthermore, routine administration of uterotonics was associated with a higher incidence of piglets presenting with meconium staining [94, 108]. Valid data on umbilical cord integrity in free farrowing systems are scarce. However, one study reported that 85% of piglets were born with an intact umbilical cord [54]. Therefore, it can currently only be hypothesized that a high proportion of piglets with ruptured umbilical cords, an observation that requires precise and continuous monitoring, may serve as an indicator of dystocia.

### Placenta traits

To date, placental characteristics in sows have been largely overlooked in both free farrowing and crated systems. In crated sows, placental expulsion is typically completed within 4–5 hours after the last piglet is born [25, 109, 110] and deviations from this timing may lead to retained placentas, which are classified as either total or partial. Total retained placentas are defined as the absence of placental expulsion during the observation period, whereas partial retained placentas occur when the last placental part is expelled before the birth of the final piglet [75]. The incidence of retained placentas in crated sows has been reported to range from 3% to 6% [75]. Interestingly, farrowing duration is significantly influenced by placental retention. Sows without retained placentas (*n* = 134) had an average farrowing duration of 369 ± 203 minutes, while those with partial retained placentas (*n* = 4) exhibited longer farrowing durations (734 ± 136 min.; *p* = 0.021). Total retained placentas (*n* = 4) were associated with the longest farrowing durations (1009 ± 275 min.; *p* = 0.004), indicating a clear association between placental retention and prolonged farrowing [75]. These findings highlight the impact of retained placentas on reproductive performance and underscore the importance of monitoring placental expulsion in sow management.

Several studies on free farrowing sows indicate that placental expulsion duration (time interval between the first placenta and the last placenta being expelled)is longer in this housing system (224 ± 181 min [53]; 233 ± 140 min [59]; 365 ± 262 min [23]; 389 ± 300 min [24]; 407 ± 292 min [54]) compared to crated systems (159 ± 99 min in gilts [52]; 241 ± 240 min [109] and 264 ± 216 min [75]). While the reasons for these differences remain unclear, it is hypothesised that the free farrowing system allows the third stage of labour to proceed more naturally, as myometrial contractions decrease in amplitude after the last piglet is expelled and may even reverse direction from the cervix to the uterine tube [111]. However, based on current knowledge, there are limited data directly linking prolonged placental expulsion to improved subsequent performance parameters. Therefore, it remains unclear whether a longer duration of placental expulsion should be interpreted as physiologically neutral, advantageous, or potentially detrimental. At present, this finding should be considered an observational characteristic of the housing system rather than evidence of a causal effect.

However, farrowing duration had a significant effect on placenta expulsion duration (duration between first and last placenta) [52, 75] as well as the onset of placental expulsion (first expulsion of placenta) [75]. Therefore, a possible key indicator for monitoring parturition in free farrowing sows is the onset of placental expulsion [24]. Sows that expelled the first placenta part before the birth of the last piglet experienced significantly longer farrowing durations compared to those that expelled the first placenta part concurrently with the last piglet or after the last piglet (305 ± 216 min vs. 139.0 ± 34.9 min; *p* < 0.01) [24]. Additionally, the number of placental parts may serve as a predictor for farrowing and placental expulsion durations in free-farrowing sows, with both durations being significantly prolonged when five or more placental parts are expelled [24]. This suggests that during prolonged farrowing, the placenta is subjected to increased mechanical stress, making it more likely to be expelled in multiple fragments. However, further studies focusing on placental expulsion duration in free farrowing sows would be required to confirm this hypothesis. Overall, it should be noted that monitoring placental expulsion in free-farrowing systems can sometimes be complicated by placentophagy, which sows may exhibit occasionally during farrowing, though it more commonly occurs after the farrowing process [112]. 

## Conclusions

In conclusion, the transition to free farrowing systems represents a major advancement in sow welfare, providing greater freedom of movement and supporting the expression of natural behaviours, particularly nest-building. The provision of adequate nesting material is essential, as it facilitates prepartum nest-building and optimizes both behavioural and endocrine preparation for parturition.

Monitoring the farrowing process in these systems is critical for identifying potential disorders. Key indicators include piglet birth intervals, timing of placental expulsion, litter-level signs such as meconium staining and umbilical cord integrity, and physiological markers like progesterone concentrations at the birth of the first piglet. Structured observation allows early detection of sows at risk, while routine or excessive use of exogenous uterotonics should be avoided, as it may cause overly strong or prolonged uterine contractions, increasing piglet mortality and reducing colostrum intake.

Placental expulsion patterns provide valuable insight into the progession of parturition. Specifically, sows that expel the first placenta before the last piglet, or that pass more than four placental parts during farrowing, are considered to have risk factors at higher risk for prolonged farrowing. Meconium scoring and umbilical cord assessment further provide practical, litter-level indicators of dystocia, as higher scores and a greater prevalence of ruptured cords are associated with farrowing complications.

Overall, the farrowing process in free farrowing systems is influenced by a complex interplay of nest-building behaviour, endocrine regulation, placental dynamics, and management practices. While these systems offer substantial welfare benefits, further research is needed to refine monitoring strategies, define evidence-based intervention thresholds, and fully harness the potential of free farrowing to optimize outcomes for both sows and piglets.

## Data Availability

No datasets were generated or analysed during the current study.
